# Dr. Clymer’s Correction

**Published:** 1842-10-08

**Authors:** 


### THE MEDICAL EXAMINER.

### PHILADELPHIA, OCTOBER 8, 1842.

We have received a communication from Dr. Clymer, in explanation of the mistake, committed by him in his paper on amputations, with reference to Dr. Hayward and the Massachusetts General Hospital. Dr. Clymer, we learn, took his data from an analysis of the combined results of the Massachusetts General Hospital and the Pennsylvania Hospital, which appeared in the Medical Examiner, Vol. 3d, p. 298, and, by mistake, he gave these combined results, in place of those of the Massachusetts Hospital alone. Dr. Hayward's report, correctly stated, was 67 amputations, of whom 15 died, and not 70, the number given by Dr. Hayward in the extract from his note which we published last week.

